# Egg vs. Oil in the Cookbook of Plasters: Differentiation of Lipid Binders in Wall Paintings Using Gas Chromatography–Mass Spectrometry and Principal Component Analysis

**DOI:** 10.3390/molecules29071520

**Published:** 2024-03-28

**Authors:** Jana Nádvorníková, Václav Pitthard, Ondřej Kurka, Lukáš Kučera, Petr Barták

**Affiliations:** 1 Department of Analytical Chemistry, Faculty of Science, Palacký University, 17. Listopadu 12, 779 00 Olomouc, Czech Republic; ondrej.kurka@upol.cz (O.K.); lukas.kucera@upol.cz (L.K.); petr.bartak@upol.cz (P.B.); 2 Conservation Science Department, Kunsthistorisches Museum, Burgring 5, 1010 Vienna, Austria; vaclav.pitthard@khm.at

**Keywords:** binding media, cholesta-3,5-dien-7-one, dicarboxylic acids, egg, gas chromatography–mass spectrometry, linseed oil, principal component analysis, P/S ratio

## Abstract

Wall paintings are integral to cultural heritage and offer rich insights into historical and religious beliefs. There exist various wall painting techniques that pose challenges in binder and pigment identification, especially in the case of egg/oil-based binders. GC-MS identification of lipidic binders relies routinely on parameters like the ratios of fatty acids within the plaster. However, the reliability of these ratios for binder identification is severely limited, as demonstrated in this manuscript. Therefore, a more reliable tool for effective differentiation between egg and oil binders based on a combination of diagnostic values, specific markers (cholesterol oxidation products), and PCA is presented in this study. Reference samples of wall paintings with egg and linseed oil binders with six different pigments were subjected to modern artificial ageing methods and subsequently analysed using two GC-MS instruments. A statistically significant difference (at a 95% confidence level) between the egg and oil binders and between the results from two GC-MS instruments was observed. These discrepancies between the results from the two GC-MS instruments are likely attributed to the heterogeneity of the samples with egg and oil binders. This study highlights the complexities in identifying wall painting binders and the need for innovative and revised analytical methods in conservation efforts.

## 1. Introduction

Wall paintings are one of the oldest forms of artistic expression and are an important part of cultural heritage. Scientific research is being carried out to determine the material composition of paintings, as well as their ageing mechanisms and restoration impacts. It is important to ensure that conservation measures preserve the historical and artistic value of the paintings. Further, research has explored painting techniques to aid in the authentication and attribution of artworks [1,2]. In recent years, there has been a significant scientific effort to develop innovative and non-invasive analytical methods for studying wall paintings. Since these paintings are often located in remote or challenging locations, portable techniques are preferred [2,3,4,5]. Optical methods such as spectroscopy and microscopy (e.g., UV-induced fluorescence photography, XRF imaging, and Raman spectroscopy) are usually used, as they enable detailed examination without direct contact [6,7,8]. However, non-invasive methods may not always be sufficient. Micro-invasive and micro-destructive approaches can provide more comprehensive analysis while minimising the impact on the artwork during sample collection [2].

There are various techniques for wall painting, varying based on the plaster condition, binders, and pigments. A fresco method involves applying pigments to fresh, wet lime plaster. On the other hand, a secco is performed on dry plaster and requires binding media such as animal glues, casein, drying oils, or eggs [9,10,11]. Determining the binders is challenging due to their complex composition, minimal sample availability (<1 mg), and the low binder-to-pigment ratio (ca. 10% *w*/*v*) [7,12,13,14]. It is also affected by external factors such as climatic conditions and air pollutants, as well as internal interactions between the binders and pigments that contribute to degradation over time [15,16,17]. Accurate identification of the binding media is crucial for historical reconstruction and restoration efforts; this requires an understanding of the degradation products and sensitive analytical techniques [18,19,20].

Eggs served as a prevalent painting binder from ancient times to the 15th century, when they gradually gave way to drying oils [12,21,22,23]. The use of linseed, poppyseed, and walnut oils became prevalent in the 16th and 17th centuries, persisting as a tradition [12,24,25]. However, many paintings used a combination of both binders, either separately on distinct sections or together in the form of an oil and egg emulsion known as “tempera grassa” [12,26]. The decision to use both binders was primarily driven by visual considerations, as certain pigments were incompatible with oil, risking yellowing due to the oil ageing [25,27,28] or producing greenish hues when combined with egg yolk [19]. Indeed, analysis of egg and oil binders side by side can be a challenging task [29].

Gas chromatography combined with mass spectrometric detection (GC-MS) is a commonly used technique for identifying organic materials, especially lipidic binding media [13,21,30]. Fatty acid methyl ester (FAME) analysis forms the basis of lipid binder identification using GC-MS [31,32]. While the palmitic-to-stearic-acid ratio (P/S) was traditionally considered a reliable parameter, which remained constant during ageing [12,13,33,34], recent studies have highlighted its susceptibility to numerous factors. These include the sample age, composition, conservation efforts, cleaning processes [24,31,35], and the faster evaporation rate of selected saturated fatty acids [36]. Caution is advised when relying solely on the P/S ratio for identification due to sample dilution and instrument response variations [37]. Alternative parameters offer more precise identification. These include the azelaic-to-palmitic-acid ratio (A/P) [7,38,39]; the azelaic-to-other-dicarboxylic-acid ratio, such as suberic (Su) or sebacic (Seb) acid—A/Su, A/Seb [35,40]; the sum of the relative content of dicarboxylic acids (ΣD) [38]; and the oleic-to-palmitic-acid or -stearic-acid ratio (O/P, O/S) [16,40,41]. Differentiating between egg and oil can be aided by the A/P ratio and ΣD. Drying oils have a higher amount of unsaturated fatty acids compared to eggs. This leads to the production of more dicarboxylic acids, which are generated through degradation (oxidative) processes from these unsaturated acids and result in notably higher values in the A/P ratio and ΣD in drying oils [16]. However, these parameters may still be influenced by the film thickness, pigment type, oil source and processing, and the painting’s storage and preservation history [13,21,42].

While GC-MS analysis of fatty acid methyl esters is a well-established practice in the literature, the use of fatty acid ratios for identifying lipidic binders still poses many challenges. Typically, the results are expressed as ratios of the chromatographic peak areas rather than in concentration units, leading to potential variations depending on the methodology employed. Consequently, the outcomes of different researchers may differ significantly, highlighting the limited reliability and accuracy of this evaluation method [43]. The purpose of this study is to demonstrate the extent of the potential discrepancies in the observed diagnostic values (A/P, A/Su, O/S, P/S, and ΣD) even when the data are obtained using identical methods and using two GC-MS instruments with nearly identical hardware configurations. Additionally, the study aims to propose a solution to this issue by employing a combination of markers based on principal component analysis. This approach is meant to highlight the issue of the substantial differences in the obtained values, as well as enable a more reliable differentiation between the egg and oil binders used in paintings. Finally, this study also stresses the importance of utilising reference samples of wall paintings for egg and oil binding media, which should undergo artificial aging to simulate historical samples and then can serve as a reliable benchmark reference for verifying the reliable diagnostic value ranges.

## 2. Results and Discussion

### 2.1. Derivatisation of the Samples

GC-MS analysis of lipidic binding media is based on the determination of fatty acids, which must first be released from the respective triacylglycerols [12]. The liberated fatty acids are converted into a suitable form for analysis, with the most common form being fatty acid methyl esters (FAMEs). Traditionally, FAMEs have been prepared via saponification followed by methylation, e.g., with diazomethane [21,44,45] or methanol under acidic catalysis [46]. The procedure of processing samples for analysis can be quite lengthy, as it involves several steps, such as hydrolysis, acidification, extraction, and derivatisation. This not only leads to a prolonged processing time but also increases the risk of sample loss or contamination during handling [47,48]. Quaternary ammonium hydroxides, especially *m*-(trifluoromethyl)phenyltrimethylammonium hydroxide (TFTMAH), have become increasingly popular, as they offer numerous advantages over other types of reagents [1,39,49]. TFTMAH is a one-step esterification/transesterification reagent that requires little to no sample preparation; thus, this procedure is faster than traditional methods. Additionally, it is less basic and does not require excessively high reaction temperatures, which reduces the likelihood of undesirable side reactions [50]. To facilitate the solubilisation of nonpolar analytes, TFTMAH was applied, together with toluene and methanol, and the mixture was heated to 60 °C, the optimal temperature for obtaining the maximum yields, as described previously [25]. Numerous fatty acids were identified in the model samples with egg and linseed oil binders (Figure S1 and Table S1 in the Supplementary Materials); however, particular attention was paid to azelaic, oleic, palmitic, sebacic, stearic, and suberic acids and their characteristic ratios (P/S, A/P, O/S, and A/Su).

### 2.2. Variations in the Diagnostic Values

The diagnostic ratios of fatty acids serve as widely recognised indicators for distinguishing between egg and oil binding media. However, the reports from the literature show significant variations in values. For instance, the reported P/S ratio ranges from 0.5 to 4.1 for egg binder [36,41] and from 1.2 to 2.0 for linseed oil [30,31]. Obviously, the variations are more pronounced in eggs due to the occurrence of specific egg components (yolk, white, or whole egg), their more complex composition, and the lower homogeneity of the samples [41]. Additionally, the parameters of the hardware instrumentation, not extensively addressed in previous studies, can influence the values. Preliminary experiments with various hardware setups indicated that the values could differ significantly depending on the used instrumentation and current conditions. Therefore, the model samples of the wall paintings were analysed using the same method in two laboratories, located in Olomouc (GC-MS 1) and in Vienna (GC-MS 2), using two nearly identical GC-MS instruments, differing only slightly in their column stationary phase and manufacturer. Both columns were nonpolar. A 5% diphenyl—95% dimethylpolysiloxane phase was used in GC-MS 1 (OPTIMA-5 MS) [51] and a phenyl arylene polymer, which is virtually equivalent to 5% diphenyl-95% dimethylpolysiloxane, was used in GC-MS 2 (DB-5ms, Low Bleed, Ultra Inert) [52].

### 2.3. Artificial Ageing

Reference samples of the wall paintings were subjected to artificial ageing to better understand the chemical changes in the composition of the lipid binders and the mechanisms that occur during ageing. These samples may experience certain mechanisms, such as photooxidative processes, cleavages, and degradation reactions, which normally occur over decades or even centuries. However, the laboratory can accelerate these processes by utilising UV light, humidity, and elevated temperatures [49,53]. Before and after each step of the artificial ageing process (NA = non-aged samples, UV1 = ageing for 504 h, UV2 = ageing for 1008 h, and UV3 = ageing for 1512 h), a group of samples was collected from a reference block of wall paintings (details are presented in the Materials and Methods section). It contained seven egg binder samples (one non-pigmented and six pigmented samples) and seven samples with a linseed oil binder (with the same pigments as in the egg samples). The diagnostic ratios of fatty acids and the sum of the relative representation of selected dicarboxylic acids—∑D (azelaic, sebacic, and suberic)—were monitored across the ageing steps, from unaged samples (NA) to the most aged (UV3). Significant differences in these parameters were observed not only between the ageing steps but also between the egg and oil binders. Table 1 shows the differences between the individual parameters in the egg and linseed oil binders. The average values and standard deviations of these parameters were calculated from the mentioned seven samples (egg/oil binder) with different pigments, with each sample injected three times in a total of twenty-one analyses.

### 2.4. Fatty Acids and Diagnostic Values

As can be seen in Table 1, the P/S ratio in the egg and linseed oil binding media samples remained remarkably consistent throughout and even prior to artificial ageing. For the linseed oil, the average values of the P/S ratios from steps NA, UV1, UV2, and UV3, as determined using GC-MS 1 and GC-MS 2, were 1.3 and 1.5, respectively. These values align closely with the range reported in the literature (1.3–1.6) [16,54,55]. In comparison, the P/S ratios calculated for the egg binder were 1.8 (GC-MS 1) and 2.8 (GC-MS 2). In the literature, the average value of the P/S ratio is reported to be around 3 [20,54], which is similar to the GC-MS 2 results. However, the GC-MS 1 results were lower; therefore, it is not recommended to rely solely on the P/S ratio for the identification of lipid binders. Instead, other characteristic ratios and markers should be considered for accurate identification.

The A/P ratio is a useful tool for distinguishing between drying oils and egg binders, as it indicates the oxidation and drying degree, which gradually increases over time [35,53]. This is demonstrated in Table 1, where the average A/P values of all the samples rose more than twofold during the ageing experiment. The values of 0.4 and 0.2 for the non-aged linseed oil samples increased to 0.8 (GC-MS 1 and GC-MS 2), which closely aligns with the values reported in the literature [31,53]. On the other hand, the fresh egg samples exhibited a significantly lower azelaic acid content compared to the linseed oil, resulting in lower A/P values. The initial values of 0.07 and 0.02 for the fresh egg samples increased to 0.2 and 0.1 (GC-MS 1 and GC-MS 2) with ageing. According to the literature [54], the A/P values of eggs are usually below 0.3, which is consistent with the obtained values. Surprisingly, the highest A/P values were not found in the most aged samples of linseed oil (UV3) but were achieved in the UV2 and UV1 samples. This may be explained by the potential evaporation of azelaic acid from the sample surface during ageing under UV light, as described in [49].

During ageing, the O/S ratio was monitored as a crucial parameter, particularly for the oils, because it characterises their maturity level [41,56]. The results reveal a significant decrease in the O/S ratio during artificial ageing, indicating a loss of oleic acid. The average values of the egg binder dropped remarkably from 2.7 and 4.0 to 0.2 and 0.05 (GC-MS 1 and GC-MS 2), while for the linseed oil, the values came down from 1.8 and 2.2 to 0.4 and 0.1 (GC-MS 1 and GC-MS 2). These findings are consistent with earlier research that indicates O/S ratios of less than 0.5 for historical artworks [54]. The results show that artificial ageing accelerates the oxidation of oleic acid, which is highly susceptible to oxidation due to its double bond. This makes oleic acid a valuable indicator of the degree of oxidation [49,53]. The most significant decrease in the O/S values was observed between the NA and UV1 steps.

The A/Su ratio is another important parameter, which is crucial for the evaluation of the pre-heating processes that can occur in linseed oil processing, such as exposure to higher temperatures during the preparation of the so-called polymerised oil [29,53]. A substantial decrease in the average values was observed during the artificial ageing. The average values for linseed oil were 3.8 and 4.2 and decreased to 1.9 and 2.1 for the analyses using GC-MS 1 and GC-MS 2, respectively. Raw oils have values greater than 6, and heat-processed oils have values in the range of 2–3 [53]. A mixture of polymerised and purified linseed oil was used to prepare the model samples, so the obtained results are in full agreement with the literature. A similar trend was observed in the egg samples, with the average values dropping from 3.1 and 4.4 to values of 1.2 and 1.4 for GC-MS 1 and GC-MS 2, respectively. The most significant decrease occurred between the NA and UV1 steps, except for the egg samples that were measured using GC-MS 1, where the final UV3 step resulted in the lowest average value of 1.2. This confirms that the artificial ageing process was highly effective, even after the first ageing step.

The last studied parameter was the sum of the relative representation of selected dicarboxylic acids (∑D), namely suberic (C8), azelaic (C9), and sebacic (C10) acid, which are the major oxidative products of C18 unsaturated fatty acids [12,33]. During the artificial ageing process, ∑D gradually increased, particularly in the linseed oil, as it contains a larger amount of unsaturated fatty acids compared to eggs. The highest values were observed after the final ageing step, UV3. The average ∑D values were found to be 25.7% and 35.4% for the linseed oil and 9.3% and 13.5% for the egg binder (GC-MS 1 and GC-MS 2, respectively). The results, which are presented in Table 1 as the average values obtained from three repeated injections of each sample, are in accordance with the data from the literature [16,43].

The fatty acid ratios and the parameter ∑D for the egg and linseed oil binding media were also compared statistically. The normality of all the data (see Supplementary Materials—Microsoft Office Excel file) was analysed graphically, as well as using the Jarque–Bera test (J–B test) and the Kolmogorov–Smirnov test (K–S test). The test criteria χ^2^_exp_ for the J–B test and *D* for the K–S test were compared to critical values for χ^2^ and *D* with a significance level α = 0.05 and degrees of freedom *v* (*v* = *n* − 1), respectively. The null hypothesis stated that the data were normally distributed (H_0_: χ^2^_exp_ (or *D*) < χ^2^_(1−α)_), and this was tested against the alternative hypothesis that the data were not normally distributed (H_1_: χ^2^_exp_ (or *D*) > χ^2^_(1−α)_). The calculated values were less than the critical values, so the null hypothesis was accepted. Then, an unpaired two-tailed Student’s *t*-test was applied to compare the results of the fatty acid ratios and the parameter ∑D between the egg and linseed oil binders using one instrument (GC-MS 1 or GC-MS 2) for each ageing step. The average values and standard deviations from Table 1 were used for this testing. The null hypothesis *H*_0_*: µ*_1_ *= µ*_2_ was tested against the alternative hypothesis *H*_0_: *µ*_1_ *≠ µ*_2_. The critical values for *t* with a significance level α = 0.05 and degrees of freedom *ν* (*ν = n*_1_ *+ n*_2_ − 2) were compared to the calculated values. Except for one sample (the A/Su ratio of the NA samples measured using GC-MS 2), the calculated values exceeded the critical values, so the null hypothesis was rejected. The *t*-test proved the statistically significant difference between the egg and linseed oil binders. Furthermore, an unpaired two-tailed Student’s *t*-test (confidence level of 95%) was used for comparison of the results obtained using the GC-MS 1 and GC-MS 2 instruments. The *t*-values suggested a statistically significant difference between the results obtained using GC-MS 1 and GC-MS 2 at the chosen level of significance except for seven values, where there was not significant difference. These were the values of A/P, A/Su, O/S (UV1) and A/Su, O/S (NA) for the linseed oil binder and A/Su (UV1 and UV2) for the egg binding media. Such an inconsistency likely stems from the inhomogeneity of the binders used and the small number of physical replicates of each sample. The results of the statistical testing are also supported by the results from the PCA (see Section 2.6).

### 2.5. Other Characteristic Markers

**Figure 1:**
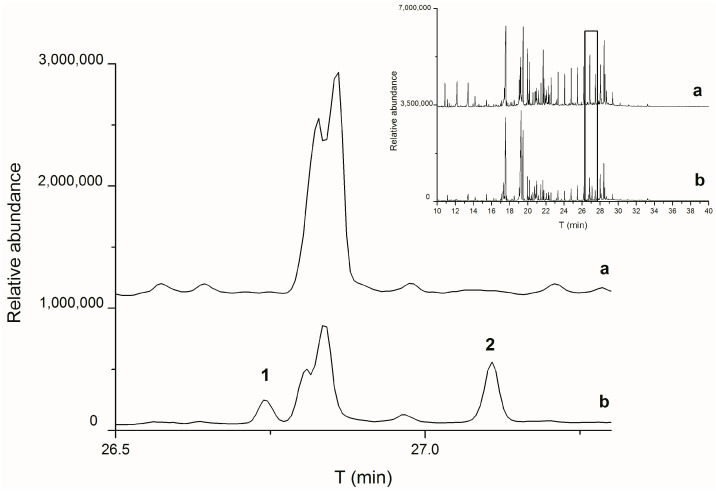
Zoomed-in total ion chromatograms (TICs) of egg samples without pigment after artificial ageing, step UV1 (a) and before ageing (b). 1—cholesterol methyl ether, 2—cholesterol. The inset shows the whole TICs of the sample before (b) and after artificial ageing (a) with a marked section of the expanded part.

Due to the described variations in the diagnostic parameters and the potential influence of various factors on the measured values, it is recommended to explore other specific substances to indicate the type of binder. Sterols, for instance, offer a promising marker group for the detection of egg binders. Cholesterol usually accounts for up to 5% of the total lipid content in eggs [57]; this is a large amount compared with the content of any sterol in a drying oil [12]. Thus, it is often considered an indicator of egg’s presence [29,43,58,59]. However, relying solely on cholesterol may yield inaccurate results due to its autooxidation [60,61], as illustrated in Figure 1. While cholesterol was initially present in all the fresh egg binder samples, it disappeared during the initial ageing step (UV1). The subsequent investigation focused on cholesterol oxidation products (COPs) and revealed various identified COPs: Cholesta-4,6-dien-3-ol, Cholesta-4,6-dien-3-one, Cholesta-3,5-diene, Cholest-5-en-3-one, and Cholesta-3,5-dien-7-one. Cholesta-3,5-dien-7-one persists even in the most aged samples (UV3), and therefore it is used as an egg binder marker in historical samples [16,62,63]. None of these COPs or cholesterol were detected in the oil binder samples. Additionally, sterols like beta-sitosterol, stigmasterol, and isofucosterol were identified in both the egg binding media and linseed oil, although the latter two are more prevalent in linseed oil [64,65]. However, they can also appear in trace amounts in eggs [57]. Correct interpretation therefore requires a quantitative evaluation of the occurrence of these substances, preferably using a suitable statistical method, such as principal component analysis.

### 2.6. PCA

A combination of diagnostic ratios of fatty acids, complemented by sterols and other markers, may not invariably suffice for the accurate identification of lipid binders. Cholesterol oxidation products emerge as potential indicators of egg binders, although it could be a challenging task to identify them in real historical samples due to their frequently low quantities, often approaching or falling below the method’s limit of detection [16]. As already mentioned, various factors significantly affect fatty acid ratios and contribute to considerable discrepancies in the measured values, which makes comparison with the reference values from the literature somewhat difficult. For instance, the P/S ratio in the model egg samples, subjected to the final stage of artificial ageing (UV3) and analysed using the GC-MS 1 instrument, exhibited average values of 1.8 ± 0.3. Such a P/S ratio value could be erroneously interpreted as indicative of linseed oil if evaluated without further context only through comparison with literary sources [20,31]. Therefore, the importance of including control samples to verify the reference values for analysis and distinguishing between egg and oil-based binders needs to be emphasised. However, obtaining authentic reference samples with a precisely documented origin and preparation methods is typically rare and rather challenging. The scarcity of such samples makes their routine use as reference standards impractical. The proposed approach, involving model samples with well-defined preparation protocols, ideally coupled with contemporary sophisticated artificial ageing procedures and techniques, represents an efficient and cost-effective strategy to address this limitation.

**Figure 2:**
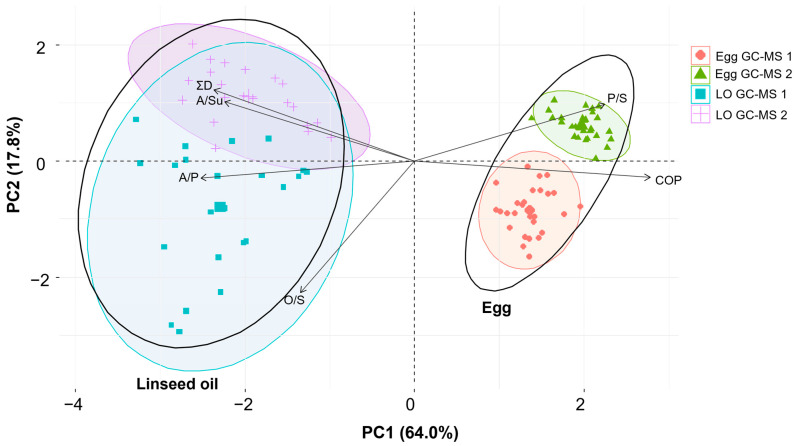
PCA biplot of egg and linseed oil (LO) binding media reference samples after artificial ageing (UV3 step) analysed using two GC-MS instruments (GC-MS 1 and GC-MS 2). The ellipsoids represent 95% confidence regions encompassing the centroids of each data cluster. Black vectors represent six variables’ loadings: A/P—azelaic/palmitic acid ratio, A/Su—azelaic/suberic acid ratio, O/S—oleic/stearic acid ratio, P/S—palmitic/stearic acid ratio, ∑D—sum of the relative representation of azelaic, sebacic, and suberic acids, COP—presence or absence of any cholesterol oxidation product.

Principal component analysis (PCA) may be an advantageous resource. This methodology is commonly used to differentiate protein binders based on the distinct representation of individual amino acids [54,66]. However, PCA has rarely been used to distinguish between lipid binders. In this study, PCA was used to evaluate the reference samples of artificially aged (UV3 step) egg and oil binders, measured using both GC-MS 1 and GC-MS 2 instruments. Six variables were considered for the PCA: the P/S, A/P, O/S, and A/Su acid ratios; the parameter ∑D; and the presence/absence of COPs. The first two principal components (PCs) contributed to 81.8% of the total variance in the data, as depicted in Figure 2. Principal components PC1 and PC2 accounted for 64.0% and 17.8% of the total variability, respectively. PC1 correlated mainly with the COPs, A/P, P/S, A/Su, and ∑D, with the strongest correlation observed between the COPs and the A/P ratio. An increase in A/P suggests an increase in A/Su and ∑D.

Further, a biplot was used to visually segregate the egg and oil binding media (black ellipses), with distinct clusters for the GC-MS 1 and GC-MS 2 instrumentation in the egg samples (red and green ellipses). This spatial distribution is likely attributed to the low homogeneity of the egg samples and consequently to a significant discrepancy in the P/S ratio (mean value 1.8 ± 0.3 for GC-MS 1 versus 2.9 ± 0.2 for GC-MS 2) and the O/S ratio (mean value 0.2 ± 0.1 for GC-MS 1 versus 0.05 ± 0.02 for GC-MS 2). On the contrary, the linseed oil samples exhibited an overlap of clusters, indicating a negligible difference in the variables analysed using the GC-MS 1 and GC-MS 2 instruments. In addition, the PCA biplot illustrated the relationship between the egg/oil binding media and six variables. The P/S ratio and the presence of COPs are significantly related to the presence of eggs, indicating that cholesterol and its oxidation products are sterols generally present only in eggs. The greater proportion of palmitic acid in animal fats than in drying oils leads to the higher P/S ratio observed in eggs [13]. In contrast, the linseed oil samples were associated with variables such as the A/P, A/Su, and O/S ratios and the parameter ∑D. This reflects the higher concentration of unsaturated fatty acids (linolenic, linoleic, oleic) and their degradation products (dicarboxylic azelaic, sebacic, and suberic acids) in oils compared to eggs [33].

## 3. Materials and Methods

### 3.1. Chemicals

Methanol and *n*-hexane of analytical grade were obtained from PENTA (Praha, Czech Republic). The toluene (analytical-grade) was purchased from Sigma-Aldrich (St. Louis, MO, USA). Further, the 3-(Trifluoromethyl)phenyltrimethylammonium hydroxide (TFTMAH), 5% *w*/*v* in methanol (known as MethPrep II), was supplied by Tokyo Chemical Industry Co., Ltd. (Nihonbashi-honcho, Chuo-ku, Tokyo, Japan). The fatty acid standards—azelaic (C9), suberic (C8), sebacic (C10), palmitic (C16:0), stearic (C18:0), oleic (C18:1), linoleic (C18:2), linolenic (C18:3), and cholesta-3,5-diene-7-one—were purchased from Sigma-Aldrich. Individual stock standard solutions were prepared for fatty acids in *n*-hexane and cholesta-3,5-diene-7-one in 2-propanol at a concentration of 1 mg·mL^−1^ and stored in a refrigerator at 5 °C.

### 3.2. Binders and Pigments

The pigments used for the preparation of the model samples were as follows—Egyptian blue, indigo, and red earth pigment (red ochre)—obtained from Zecchi (Florence, Italy). The azurite, lead-tin yellow, and ochre were purchased from Kremer Pigmente GmbH & Co. KG (Aichstetten, Germany). The oil binding media was prepared from a mixture of polymerised linseed oil (Umton Colours, Děčín, Czech Republic) and purified linseed oil (H. Schmincke & Co.-GmbH & Co. KG, Erkrath, Germany). A fresh hen egg was purchased from a local market. Only the egg yolk, meticulously separated from the egg white, was utilised as a binder.

### 3.3. Preparation of the Reference Wall Painting Samples

**Figure 3:**
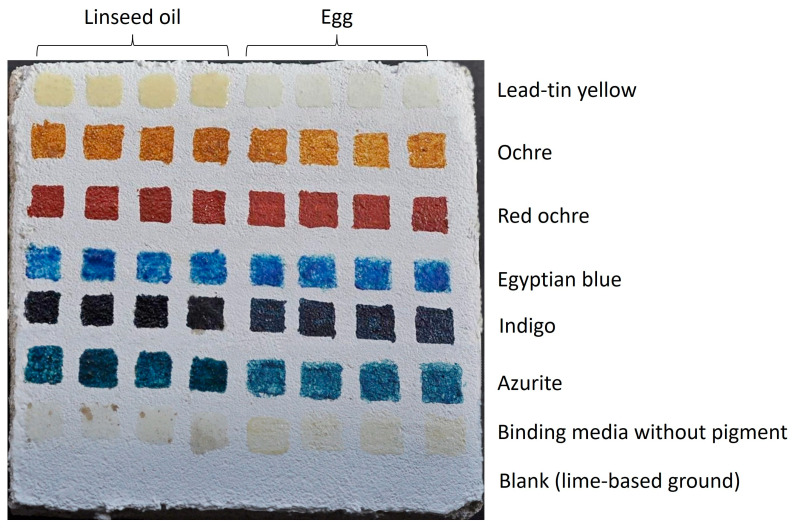
Lime-based plaster block with squares (1 cm^2^) of linseed oil and egg binding media with six various pigments.

To simulate the historical samples and study the alteration mechanisms, lime plaster blocks were prepared according to the traditional historical recipes [67]. The base coat was made of one part white slaked lime (Vápenka Čertovy schody, Tmaň Czech Republic) and three parts white coarse silica sand (Tomi-písek s.r.o., Prague, Czech Republic). Then, the blocks were left to dry in a humid environment for four days. After the base coat matured, the surface was scratched with lines to remove any loose grains and to give a key for the finish coat. The finish coat was composed of one part slaked lime and two parts fine sharp sand (particle sizes up to 2 mm) and applied thinly on top of the base coat. The surface was smoothed with a felt float trowel and a felt brush (cat’s tongue shape) and lightly dampened with water during the scouring process. Dry powdered pigments were hand-ground into the binding media on glass slides with a spatula and applied as approx. 1 cm^2^ squares onto dry lime plaster (Figure 3). Samples (squares) of the lime plaster mixed with the binding media and pigment were taken from the block three days after drying and labelled as non-aged (NA). Then, the block underwent accelerated artificial ageing.

### 3.4. Accelerated Artificial Ageing

The artificial ageing was performed using a SOLARBOX 3000e RH chamber (CO.FO.ME.GRA., Milano, Italy) equipped with a xenon lamp, which replicates the total optical spectrum of the Sun. The parameters of ageing were set as follows: Xe lamp irradiation of 500 W·m^−2^, temperature of 30 °C, and relative humidity of ~75%. The block of lime plaster with the binders and pigments was kept under these conditions for 504 h (UV1). Afterwards, one aliquot part of the reference material was put into a glass vial and stored at −20 °C prior to the GC-MS analyses. The rest of the reference samples were placed back into the SOLARBOX chamber and aged repeatedly for 504 h twice (UV2, UV3) with a total duration of 1512 h.

### 3.5. Sample Preparation

A small amount of the reference material (approximately 0.1–0.2 mg) was placed into an autosampler vial with a glass conical insert and treated with 20 µL of derivatisation mixture. It consisted of one part 5% *w*/*v* methanolic solution of Meth-Prep II and two parts solvent mixture (methanol:toluene, 1:2, *v*/*v*). The resulting mixture was vortexed for 30 s, followed by sonication for 10 min, and then heated to 60 °C for 1 h. After cooling to laboratory temperature, the samples were centrifuged for 5 min at 4400 rpm. The supernatants were transferred into a new vial with conical inserts and analysed using GC-MS. A group of samples containing seven egg binder samples (one non-pigmented and six pigmented samples, including azurite, Egyptian blue, indigo, red ochre, ochre, and lead-tin yellow) and seven samples with a linseed oil binder (with the same pigments as in the egg) were analysed at each step of artificial ageing process (NA, UV1, UV2, and UV3). For each sample, there were three replicate injections, resulting in a total of 21 analyses for egg and 21 analyses for oil binder for each step of artificial ageing.

### 3.6. GC-MS Instrumentation and Conditions

The gas chromatographic analyses were carried out using two GC-MS instruments which were situated in Olomouc (Czech Republic) and in Vienna (Austria) and were of the same type—Agilent 6890, coupled with a mass selective detector (MSD) Agilent 5973N (Agilent, Santa Clara, CA, USA). The capillary columns OPTIMA-5 MS, 30 m × 0.25 mm × 0.25 μm (Macherey-Nagel, Düren, Germany), and J&W DB-5ms UI, 30 m × 0.25 mm × 0.25 μm (Agilent, Santa Clara, CA, USA), were operated with helium (5.5, Siad, Bergamo, Italy) as the carrier gas at a constant flow rate of 0.9 mL·min^−1^. The oven program was as follows: 50 °C for 1 min, 10 °C/min ramp to a final temperature of 320 °C, and held for 12 min. The total run time was 40.00 min. A 2 μL aliquot of the derivatised extract was injected in pulsed splitless mode (206.8 kPa, 18 s, 300 °C). Identification of the compounds was based on a comparison of the respective mass spectra of the analysed compounds with the spectra of analytical standards or with the spectra in NIST Library 2020 (National Institute of Standards and Technology, Gaithersburg, MD, USA).

### 3.7. Data Analysis

The statistical software QC.Expert 3.2 (TriloByte Ltd., Pardubice, Staré Hradiště, CZ) was used for verification of the data normality, which was checked visually (histogram and Q-Q plot) and using statistical tests: the Jarque–Bera test and the Kolmogorov–Smirnov test (K–S test). The Jarque–Bera test is based on the sample skewness and kurtosis [68,69], while the K–S test is based on the empirical distribution function (ECDF) [70,71]. The software was also used for comparison of the obtained values for the egg and linseed oil binding media using an unpaired two-tailed Student’s *t*-test. This *t*-test was also used for comparison of the results obtained using the GC-MS 1 and GC-MS 2 instruments.

The GC-MS data acquired underwent analysis through unsupervised principal component analysis (PCA), incorporating univariate scaling. The resulting transformed data matrix was then transferred to the R statistical software (version 4.1.2, R Core Team 2021, Vienna, Austria), a freely available statistical computing environment. The default package “stats” was employed for conducting the PCA and facilitating visualisation. The results were evaluated by generating a biplot and employing k-means clustering to group the data into *k* clusters. The clusters are represented by 95% confidence intervals around their center (centroid), which correspond to the means of the points assigned to each cluster.

## 4. Conclusions

GC-MS stands as a well-established technique for lipid binder identification, relying on the diagnostic fatty acid ratios P/S, A/P, A/Su, and O/S and specific parameters (∑D and COP). The P/S ratio, historically crucial for distinguishing between egg and oil binders, faces scrutiny due to potential influences from various factors. In this study, reference samples of wall paintings with oil and egg binders and six different pigments were analysed using two GC-MS instruments with minor hardware differences (type of stationary phase), aiming to assess the parameter variations. The impact of artificial ageing on the model samples was also investigated. While most of the parameters changed as expected during ageing, the P/S ratio remained nearly constant. Ageing induced a decrease in unsaturated fatty acids and the emergence of dicarboxylic acids (azelaic, sebacic, and suberic), which are the oxidation products of these unsaturated acids. Both binder types exhibited altered values in parameters such as a rise in the A/P ratio and ∑D and a decrease in O/S and A/Su. A notable discrepancy in the P/S values of the aged egg binder between GC-MS 1 (1.8 ± 0.3) and GC-MS 2 (2.9 ± 0.2) prompted the exploration of additional markers, leading to the identification of cholesterol oxidation products, notably cholesta-3,5-dien-7-one, specific to eggs. However, the identification of such markers is challenging concerning the amount of the samples, the binder-to-pigment ratio, and especially the initial amount of cholesterol from which these products are subsequently created. Consequently, PCA, integrating multiple parameters, proved effective in distinguishing between the oil and egg binders, even when the P/S values deviated from the literature references. In conclusion, this study emphasises the complexities of identifying wall painting binders, particularly lipidic ones, and the importance of alternative markers and innovative analytical methods for comprehensive analysis and conservation efforts. The application of PCA provides a promising avenue for effective differentiation between egg and oil binders.

## Figures and Tables

**Table 1:** Changes in fatty acid ratios and the sum of the relative representation of dicarboxylic acids (∑D) during artificial ageing. Differences are observed between the reference samples with linseed oil and egg binding media and between the instrumentation GC-MS 1 and GC-MS 2. The average value of the selected ratio (or parameter ∑D) was calculated from seven samples of reference wall painting, each injected three times (*n* = 21). Standard deviations for each fatty acid ratio and parameter ∑D were calculated in the same way.

	GC-MS 1				GC-MS 2			
LO	NA	UV1	UV2	UV3	NA	UV1	UV2	UV3
P/S	1.3 ± 0.1	1.3 ± 0.2	1.3 ± 0.1	1.3 ± 0.1	1.4 ± 0.1	1.5 ± 0.1	1.5 ± 0.1	1.5 ± 0.1
A/P	0.4 ± 0.1	0.7 ± 0.3	0.9 ± 0.3	0.8 ± 0.3	0.2 ± 0.1	0.8 ± 0.1	0.5 ± 0.1	0.5 ± 0.2
O/S	1.8 ± 0.4	0.4 ± 0.1	0.3 ± 0.1	0.4 ± 0.3	2.2 ± 0.5	0.3 ± 0.1	0.19 ± 0.06	0.11 ± 0.07
A/Su	3.8 ± 1.1	1.8 ± 0.4	1.9 ± 0.4	1.9 ± 0.3	4.2 ± 1.3	2.1 ± 0.4	2.4 ± 0.4	2.1 ± 0.4
∑D	9.0 ± 3.5	13.4 ± 3.2	22.5 ± 3.3	25.7 ± 4.8	6.0 ± 2.6	29.5 ± 4.7	31.3 ± 7.3	35.4 ± 4.0
Egg	NA	UV1	UV2	UV3	NA	UV1	UV2	UV3
P/S	1.9 ± 0.2	1.8 ± 0.3	1.8 ± 0.3	1.8 ± 0.3	2.8 ± 0.4	2.9 ± 0.3	2.7 ± 0.3	2.9 ± 0.2
A/P	0.07 ± 0.01	0.2 ± 0.1	0.2 ± 0.05	0.2 ± 0.1	0.02 ± 0.01	0.12 ± 0.03	0.1 ± 0.04	0.13 ± 0.03
O/S	2.7 ± 0.3	0.2 ± 0.1	0.2 ± 0.05	0.2 ± 0.1	4.0 ± 0.8	0.05 ± 0.03	0.04 ± 0.02	0.05 ± 0.02
A/Su	3.1 ± 0.6	1.4 ± 0.2	1.3 ± 0.2	1.2 ± 0.1	4.4 ± 0.6	1.3 ± 0.2	1.4 ± 0.2	1.4 ± 0.3
∑D	2.0 ± 0.8	7.4 ± 3.1	7.9 ± 2.7	9.3 ± 2.1	0.9 ± 0.7	12.2 ± 1.9	13.5 ± 1.7	13.5 ± 3.1

∑D—sum of the relative representation of azelaic, sebacic, and suberic acid (%), LO—linseed oil, GC-MS 1—instrument located in Olomouc, GC-MS 2—instrument located in Vienna, NA—non-aged samples, UV1, UV2, UV3—steps of artificial ageing process.

## Data Availability

Data will be made available on request.

## Supplementary Materials

The following supporting information can be downloaded at https://www.mdpi.com/article/10.3390/molecules29071520/s1. Figure S1: Total ion chromatogram (TIC) of linseed oil sample with indigo pigment after artificial ageing (UV3). Identified fatty acid methyl esters are numbered (1-21), and degradation products of indigotin (A, B, and D) and indirubin (C) are alphabetically designated (A–D). The compounds are listed in Table S1; Table S1: Fatty acids and degradation products of indigotin and indirubin were identified in the gas chromatogram of the artificially aged (UV3) linseed oil sample with indigo pigment. The same compounds were also identified in the egg binder. The identification of compounds was based on a comparison with the corresponding mass spectra in the NIST library and with a study [72].
