# Supplementary material for: Egg vs. Oil in the Cookbook of Plasters: Differentiation of Lipid Binders in Wall Paintings Using Gas Chromatography–Mass Spectrometry and Principal Component Analysis
Source: Molecules. 2024 Mar 28;29(7):1520. doi: 10.3390/molecules29071520 (PMC11013410; doi:10.3390/molecules29071520)
Supplement: Supplementary file 1 [file molecules-29-01520-s001.zip › Supplementary-material_Molecules_Nádvorníková_REVISED.pdf]

SUPPLEMENTARY MATERIAL

Egg vs. Oil in the Cookbook of Plasters: Differentiation of Lipid Binders in Wall Paintings Using Gas Chromatography–Mass Spectrometry and Principal Component Analysis

Jana Nádvorníková [1,*], Václav Pitthard [2], Ondřej Kurka [1], Lukáš Kučera [1] and Petr Barták [1]

[1] Department of Analytical Chemistry, Faculty of Science, Palacký University, 17. listopadu 12, 779 00 Olomouc, Czech Republic
[2] Conservation Science Department, Kunsthistorisches Museum, Burgring 5, 1010 Vienna, Austria
* Correspondence: jana.nadvornikova02@upol.cz

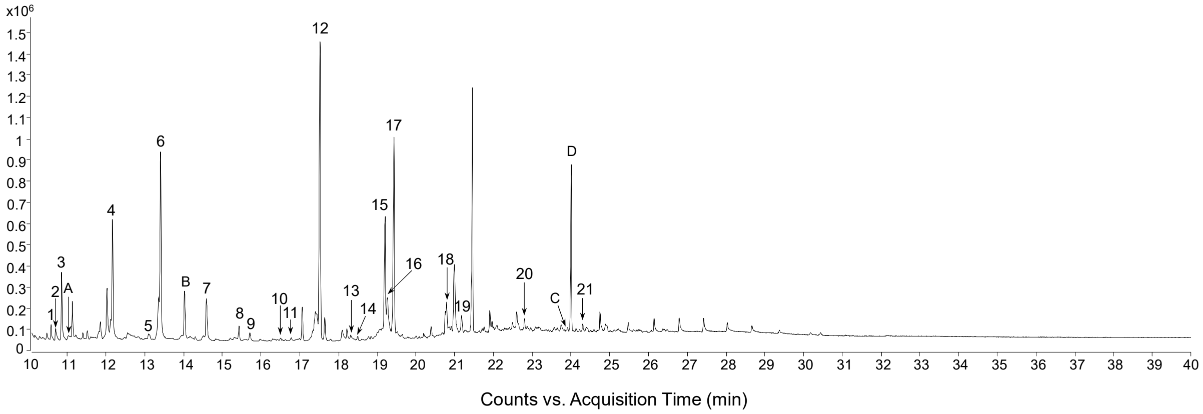

Figure S1. Total ion chromatogram (TIC) of linseed oil sample with indigo pigment after artificial ageing (UV3). Identified fatty acid methyl esters are numbered (1-21) and degradation products of indigotin (A, B, and D) and indirubin (C) are alphabetically (A-D). The compounds are listed in Table S1.

Table S1. Fatty acids and degradation products of indigotin and indirubin were identified in the gas chromatogram of the artificially aged (UV3) linseed oil sample with indigo pigment. The same compounds were also identified in the egg binder. The identification of compounds was based on a comparison with the corresponding mass spectra in the NIST library and with a study [72].

Label	Retention time (min)	Compound
1	10.577	Decanoic acid, methyl ester (capric acid)
2	10.689	Methyl 8-oxooctanoate
3	10.848	Heptanedioic acid, dimethyl ester (pimelic acid)
4	12.165	Octanedioic acid, dimethyl ester (suberic acid)
5	13.083	Decanoic acid, 9-oxo-, methyl ester
6	13.410	Nonanedioic acid, dimethyl ester (azelaic acid)
7	14.585	Decanedioic acid, dimethyl ester (sebacic acid)

8	15.427	Tetradecanoic acid, methyl ester (myristic acid)
9	15.700	Undecanedioic acid, dimethyl ester
10	16.499	Pentadecanoic acid, methyl ester
11	16.763	Dodecanedioic acid, dimethyl ester
12	17.538	Hexadecanoic acid, methyl ester (palmitic acid)
13	18.280	4-Oxododecanedioic acid, 2Me derivative
14	18.491	Heptadecanoic acid, methyl ester (margaric acid)
15	19.208	cis-9-Octadecenoic acid, methyl ester (oleic acid)
16	19.259	trans-9-Octadecenoic acid, methyl ester (elaidic acid)
17	19.447	Octadecanoic acid, methyl ester (stearic acid)
18	20.789	Oxiraneoctanoic acid, 3-octyl-, methyl ester
19	21.177	Eicosanoic acid, methyl ester (arachidic acid)
20	22.793	Docosanoic acid, methyl ester (behenic acid)
21	24.297	Tetracosanoic acid, methyl ester (lignoceric acid)
A	11.020	Methyl anthranilate
B	14.023	Methyl 2-(methoxycarbonyl)phenylmethylcarbamate
C	23.324	3-methoxy-2-(2-methoxy-1-methyl-1H-indol-3-yl)-1-methyl-1H-indole
D	24.064	2-bis-(N-methylindole-3-methoxy)
